# Validation of *in vivo* 2D Displacements from Spiral Cine DENSE at 3T

**DOI:** 10.1186/1532-429X-17-S1-Q120

**Published:** 2015-02-03

**Authors:** Gregory J Wehner, Jonathan D Suever, Christopher M Haggerty, Linyuan Jing, David Powell, Sean M Hamlet, Jonathan D Grabau, Dimitri Mojsejenko, Xiaodong Zhong, Frederick H Epstein, Brandon K Fornwalt

**Affiliations:** 1 Biomedical Engineering, University of Kentucky, Lexington, KY, USA; 2 MR R&D Collaborations, Siemens Healthcare, Atlanta, GA, USA; 3 Biomedical Engineering, University of Virginia, Charlottesville, VA, USA; 4 Pediatrics, University of Kentucky, Lexington, KY, USA; 5 Electrical Engineering, University of Kentucky, Lexington, KY, USA

## Background

Displacement Encoding with Stimulated Echoes (DENSE) is a cardiac magnetic resonance technique that encodes tissue displacement into the phase of the magnetic resonance signal. Due to the stimulated echo acquisition, the signal to noise ratio is low and fades through the cardiac cycle due to T1 relaxation. To compensate, a spiral cine DENSE sequence has been developed and used at 1.5T. This spiral sequence has not been validated at 3T, where increased field inhomogeneities and off resonance effects may result in measurement errors. We hypothesized that spiral cine DENSE is valid at 3T and tested this hypothesis by measuring displacement errors at both 1.5T and 3T *in vivo*.

## Methods

**Figure 1:**
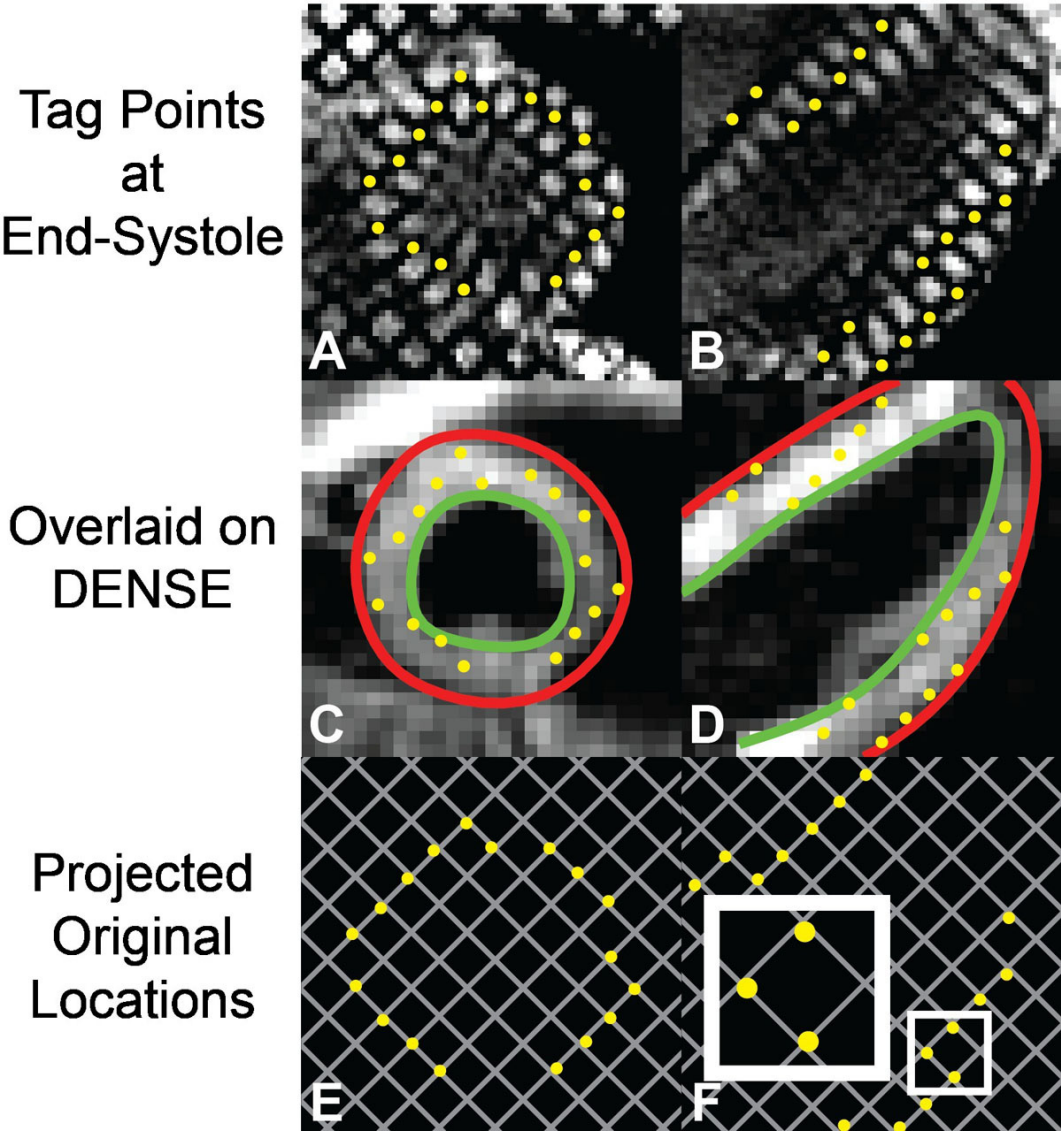
DENSE displacements project tag intersection points back into a perfect grid. By end-systole, a perfect grid of tag intersection points has deformed into a warped grid as seen in the first row for both a short-axis and four-chamber slice (A,B). The deformed intersection points can be overlaid on DENSE images taken at the same point in the cardiac cycle (C,D). The Eulerian displacements from the DENSE images can be used to project the tag intersection points back into a nearly perfect grid (E,F). Deviation from a perfect grid of 8x8 mm^2^ is a measure of the error in DENSE displacements. The small box in F is enlarged to show an example of the small deviations from the nearest grid intersections.

2D Spiral cine DENSE and tagged imaging of the left ventricle were performed on 10 healthy subjects at 3T and 6 healthy subjects at 1.5T. The tagged images were acquired with 8mm tag spacing, and were used to define the locations of tag intersection points near end-systole (Figure 1A,1B). The displacements within the DENSE images were used to project the intersection points back into a nearly perfect grid (Figure 1E,1F). The deviation from a perfect grid was used as a measure of accuracy and quantified as root-mean-squared error (RMSE) between the projected points and the perfect tag grid. This measure of accuracy was compared between 3T and 1.5T with the Wilcoxon rank sum test. Inter-observer variability of peak strains and torsion quantified by DENSE and agreement between DENSE and HARP-based analysis of the tagged images were also assessed by Bland-Altman analyses and coefficient of variation. DENSE acquisition parameters were the same at both field strengths: 6 spiral interleaves, 1 average, FOV=360x360 mm^2^, matrix=128x128, slice thickness=8 mm, TE/TR=1.08/17 ms, flip angle=20°, navigator acceptance window=±3mm. At 3T, a cardiac-gated field map was acquired during a 10 second breath-hold and used for shimming during the DENSE and tagged acquisitions.

## Results

**Figure 2:**
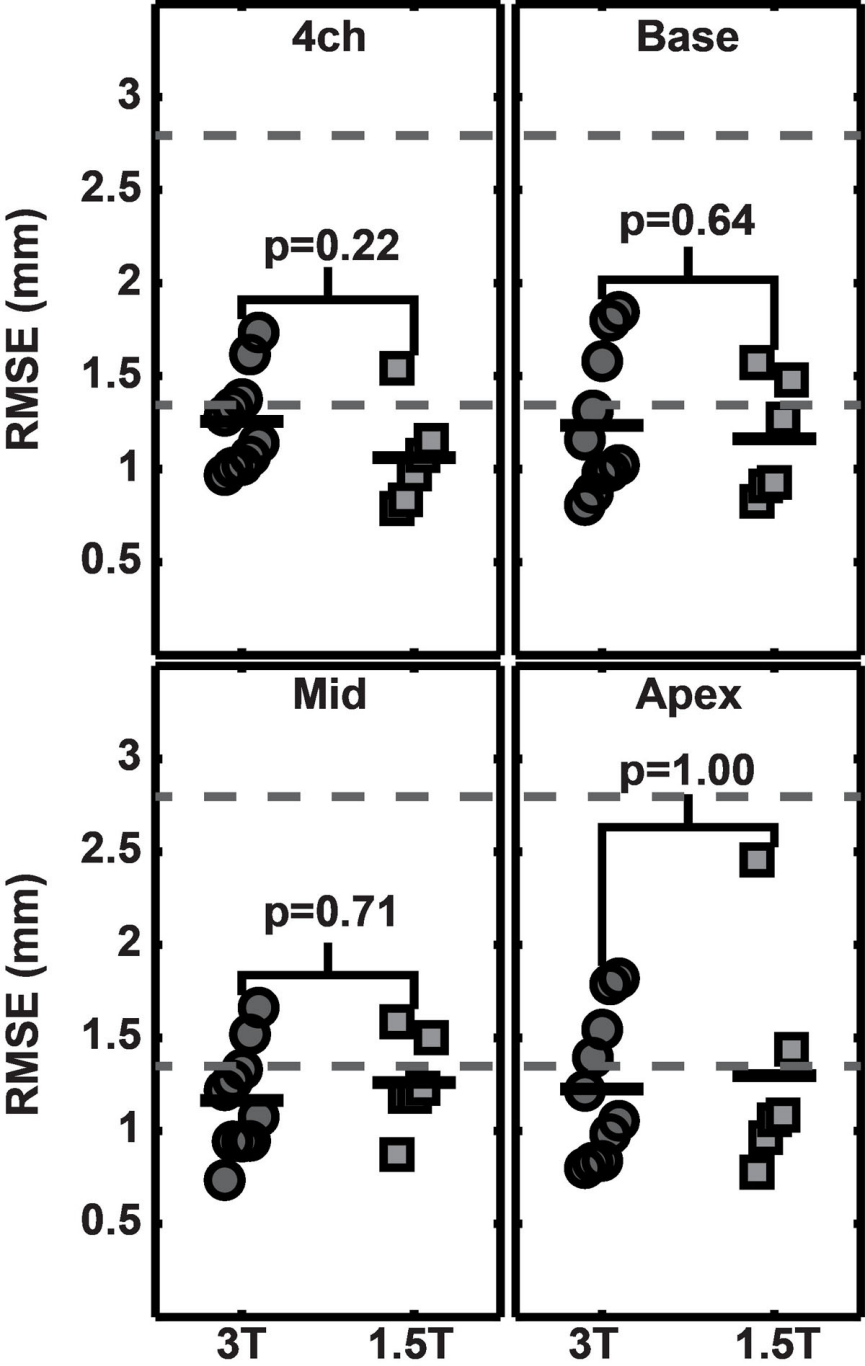
RMSE for each slice type at 3T and 1.5T. The error in DENSE displacements as measured by RMSE is shown for each type of slice. The top gray line indicates the DENSE pixel spacing of 2.8 mm. The bottom gray line is placed at 1.35 mm, which is the average of the tag pixel spacing at 3T and 1.5T (1.3 mm and 1.4 mm, respectively). The mean RMSEs are below the DENSE pixel spacing and are below or on the order of the tagged pixel spacing. No significant difference in RMSE were seen between 3T and 1.5T by the Wilcoxon rank sum test for any slice location. 4ch - Four-chamber.

The displacement accuracy in spiral cine DENSE at 3T was not different from the displacement accuracy at 1.5T (Figure 2). Across the four slice types (four-chamber, base, mid, apex), the RMSEs at 3T and 1.5T were 1.2 ± 0.3 mm and 1.2 ± 0.4 mm, respectively. Both values were significantly lower than the DENSE pixel spacing of 2.8 mm. There were no substantial differences in inter-observer variability of DENSE or agreement of DENSE and HARP between 3T and 1.5T for any of the peak strains (circumferential, radial, and longitudinal) or torsion. Through the cardiac cycle, the SNR at 3T was greater than the SNR at 1.5T by a factor of 1.4 ± 0.3.

## Conclusions

The same spiral cine DENSE acquisition that has been used at 1.5T for quantification of cardiac displacements can be applied at 3T with equivalent accuracy. The inter-observer variability and agreement of DENSE-derived peak strains and torsion with HARP is also comparable at both field strengths. Future studies with spiral cine DENSE may take advantage of the additional SNR at 3T.

## Funding

This work was supported by a National Institutes of Health (NIH) Director's Early Independence Award (DP5 OD-012132), NIH grant number T32 HL-072743, and NIH grant numbers UL1TR000117 and KL2 RR033171 from the National Center for Research Resources and the National Center for Advancing Translational Sciences. The content is solely the responsibility of the authors and does not necessarily represent the official views of NIH.

